# An AI-driven clinical care pathway to reduce 30-day readmission for chronic obstructive pulmonary disease (COPD) patients

**DOI:** 10.1038/s41598-022-22434-3

**Published:** 2022-11-30

**Authors:** Lin Wang, Guihua Li, Chika F. Ezeana, Richard Ogunti, Mamta Puppala, Tiancheng He, Xiaohui Yu, Solomon S. Y. Wong, Zheng Yin, Aaron W. Roberts, Aryan Nezamabadi, Pingyi Xu, Adaani Frost, Robert E. Jackson, Stephen T. C. Wong

**Affiliations:** 1 AI in Medicine Group, Systems Medicine and Bioengineering Department, Houston Methodist Cancer Center, 6670 Bertner Ave, Houston, TX 77030 USA; 2 Department of Neurology, Guangdong Second People’s Hospital, Guangzhou, 510317 Guangdong People’s Republic of China; 3 Internal Medicine Department, Mayo Clinic Health System, Mankato, MN 56001 USA; 4 Baylor University School of Law, Waco, TX 76706 USA; 5 T.T. & W.F. Chao Center for BRAIN, Houston Methodist Hospital, Houston, TX 77030 USA; 6 Division of Maternal Fetal Medicine, Department of Obstetrics, Gynecology and Reproductive Sciences, Houston McGovern Medical School, University of Texas Health Science Center, Houston, USA; 7 Department of Medicine, Houston Methodist Hospital, Houston, TX 77030 USA; 8 Department of Neurology, The First Affiliated Hospital of Guangzhou Medical University, Guangzhou, 510120 Guangdong People’s Republic of China; 9 Houston Methodist Research Institute, Houston Methodist Academic Institute, Houston Methodist Hospital, Houston, TX 77030 USA; 10 Department of Medicine, Houston Methodist Hospital and Weill Cornell Medicine, Houston, TX 7730 USA; 11 Department of Radiology and Houston Methodist Cancer Center, Houston Methodist Hospital, Weill Cornell Medicine, Houston, TX 77030 USA

**Keywords:** Health services, Risk factors, Chronic obstructive pulmonary disease, Disease prevention

## Abstract

Healthcare regulatory agencies have mandated a reduction in 30-day hospital readmission rates and have targeted COPD as a major contributor to 30-day readmissions. We aimed to develop and validate a simple tool deploying an artificial neural network (ANN) for early identification of COPD patients with high readmission risk. Using COPD patient data from eight hospitals within a large urban hospital system, four variables were identified, weighted and validated. These included the number of in-patient admissions in the previous 6 months, the number of medications administered on the first day, insurance status, and the Rothman Index on hospital day one. An ANN model was trained to provide a predictive algorithm and validated on an additional dataset from a separate time period. The model was implemented in a smartphone app (Re-Admit) incorporating four input risk factors, and a clinical care plan focused on high-risk readmission candidates was then implemented. Subsequent readmission data was analyzed to assess impact. The areas under the curve of receiver operating characteristics predicting readmission with ANN is 0.77, with sensitivity 0.75 and specificity 0.67 on the separate validation data. Readmission rates in the COPD high-risk subgroup after app and clinical intervention implementation saw a significant 48% decline. Our studies show the efficacy of ANN model on predicting readmission risks for COPD patients. The AI enabled Re-Admit smartphone app predicts readmission risk on day one of the patient’s admission, allowing for early implementation of medical, hospital, and community resources to optimize and improve clinical care pathways.

## Introduction

In 2012, the Centers for Medicare and Medicaid Services (CMS) launched the Hospital Readmissions Reduction Program (HRRP) to reduce the risk of readmission in patients hospitalized for acute myocardial infarction, pneumonia, and heart failure. In 2014, it was extended to include readmissions for elective total knee and total hip replacements as well as chronic obstructive pulmonary disease (COPD) exacerbations^1^. Based on 2013–2016 Medicare data, the 30-day observed readmission rate for pneumonia was 16.9%, and for COPD was 19.8% from 2014 to 2015^2^. A 30-day rehospitalization rate of 17.6% among Medicare beneficiaries in 2005 equated to an estimated expense of $17 billion^3^.

The Department of Health and Human Services identified effective transition of care as a major quality improvement goal^4,5^. To reduce unnecessary readmissions and identifiy high readmission risk patients early, providers should facilitate timely post-discharge support. However, readmission risk models historically have performed poorly^6,7^.

The objectives of this study are four-fold: (1) to extract day one admission data from a clinical data warehouse and apply machine learning techniques to develop a readmission risk prediction app for patients hospitalized with the primary diagnoses of “COPD”. The app will utilize a minimal number of input variables available on day one of the hospitalization; (2) to validate the app in a second population; (3) to initiate in-hospital interventions and early post discharge care planning, based on the app’s designation of ‘high risk of readmission’; and (4) to prospectively evaluate the subsequent readmission rates.

## Results

### Parameter selection and data characteristics

**Figure 1:**
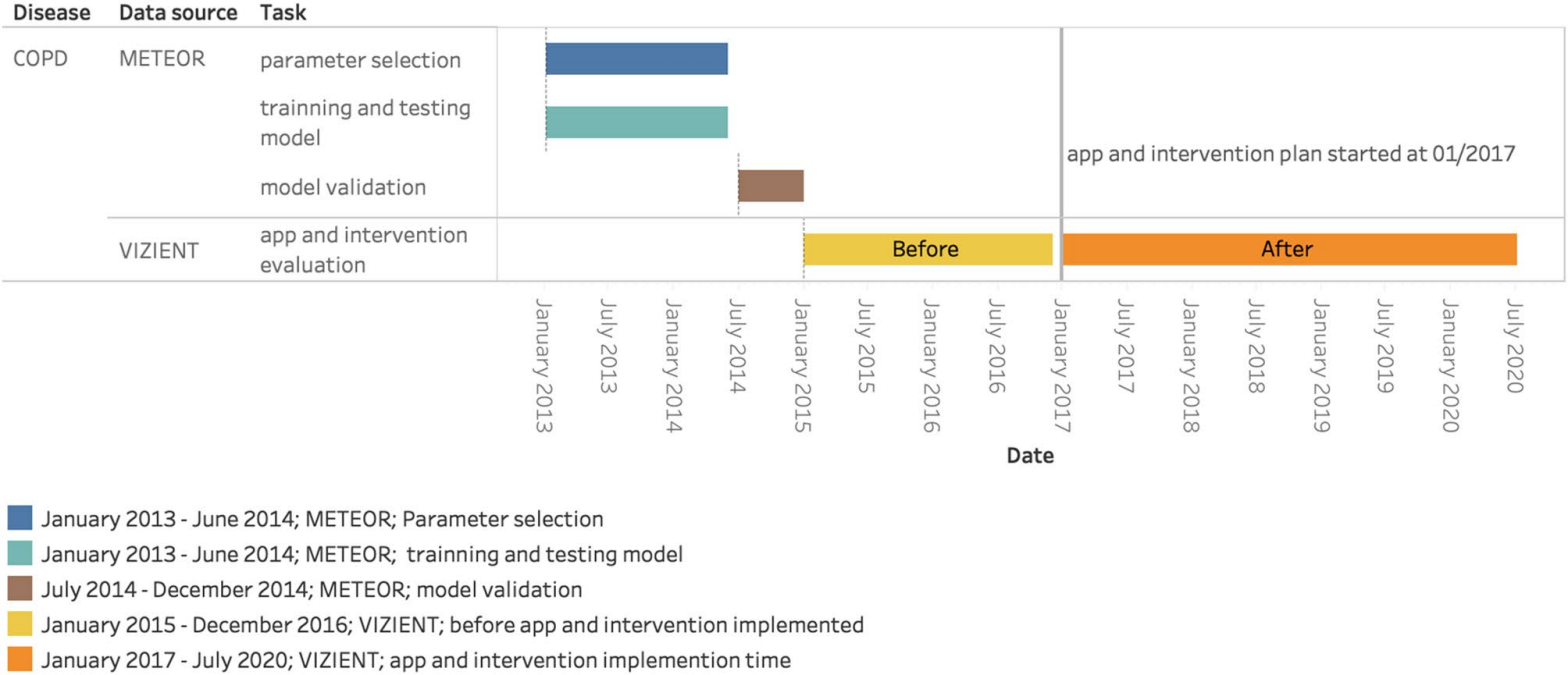
The time period and data source of collected data for each task in the process of model developing.

Figure 1 shows the time period and data source of the data collected for each task in the model development process. Variables for univariate analysis were derived from 3005 COPD patients from January 2013 to June 2014, except Rothman Index. Supplementary Tables S1 outlines the results of the univariate analysis for each parameter among those patients. 332 COPD patient visits from January 2013 to June 2014 were collected as training sample (with Rothman Index). Four parameters highly predictive (*p* value < 0.01) of subsequent readmission, identified by the Wald test include: the number of in-patient admissions in the previous 6 months, number of medications administered on admission day, insurance status, and Rothman Index on hospital day one.

A summary of the training dataset is provided in Table 1 and Supplementary Table S2. The raw dataset was pre-processed prior to modeling using our machine learning algorithm. The ‘Rothman Index at the day of admission’ numerical score was binned into four groups and labeled as 0–3 with those cut points that maximize the difference of the readmission ratio between the groups^8^. The variable ‘insurance status’ has five categories: “Medicare”, “Managed Care”, “Self-Pay”, “Other” and “unknown”, and was recoded with indicator variables. This retrospective dataset forms the basis of the information used to train and develop the artificial neural network and logistic regression model’s predictive capabilities. A separate prospective dataset was then used for validation. The relative importance of the input variables was given in Supplementary Tables S3 calculated by using the Garson’s algorithm^9^.

**Table 1:** Full dataset, Characteristics, and variables used in readmission prediction of COPD patients.

**COPD**	
Total number	332
Percent readmission within 30 days	41.6%
Number of in-patient visits within prior 6 months, mean (2.5%, 25%, 50%, 75%, 97.5% quantiles)	0.9 (0, 0, 0, 1, 5)
Number of unique medications at admission day, mean (2.5%, 25%, 50%, 75%, 97.5% quantiles)	10.5 (1, 6, 10, 14, 21)
Insurance status	
Medicare/MGD Care/Other/Self-pay/Unknown (%)	80.1/9.0/2.1/1.8/6.9
Rothman Index at the day of admission, mean (2.5%, 25%, 50%, 75%, 97.5% quantiles)	72.0 (35.1, 63.0, 75.6, 83.3, 91.3)

### Validation of the models on the separate data

The final artificial neural network model achieved an average AUC of 0.683 (± 0.009) by three-fold cross-validation on the training data, while the average AUC for the logistic regression model was 0.640 (± 0.038), demonstrating that the artificial neural network model has better prediction power over the logistic regression model for readmission for COPD patients.

**Figure 2:**
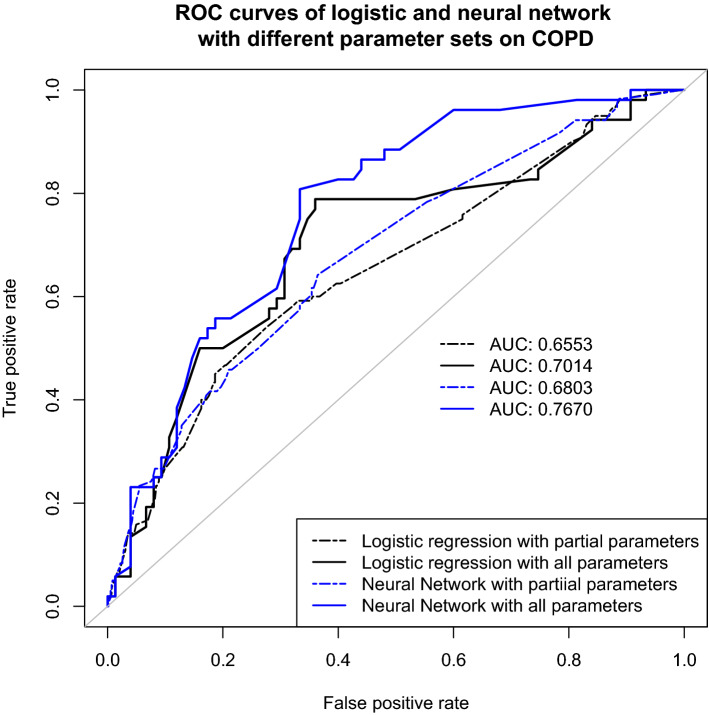
Comparisons of Logistic Regression and Artificial Neural Networks models by the receiver operating characteristic (AUCROC) curve for COPD validation samples. Partial parameters include the number of in-patient admissions in the previous 6 months, number of medications prescribed on admission day, insurance status; Rothman Index is added to form all parameters we used in the model.

The predictive results were validated prospectively in 172 patients with COPD (July 2014–December 2014) before the implementation of clinical plan intervention. AUCROC curves were compared to evaluate the predictive power of the two models on the COPD validation samples (Fig. 2). We also trained a logistic regression model and an artificial neural network model on the 3005 COPD patients, using partial parameters without Rothman Index, which was validated on 705 patients with COPD (July 2014–December 2014). The two dashed ROC curves indicate the performances of the two models without Rothman Index. The AUCROC of the COPD validation data result without Rothman Index was 0.655 for logistic regression and 0.680 for artificial neural network; the AUCROC of the COPD validation data result with all four parameters was 0.701 for logistic regression and 0.767 for artificial neural network. The difference between the validation results suggests that even with using different parameters, the artificial neural network has better prediction performance for readmission than logistic regression for COPD patients. The artificial neural network model using the four parameters, with percentile score 50% as the threshold for high-risk patients, achieves a sensitivity 0.75 (95% CI 0.61–0.86), specificity 0.67 (95% CI 0.55–0.77), and positive predictive value (PPV) 0.61 (95% CI 0.48–0.73) on the validation data. The calibration curves demonstrate poorer alignment (accuracy) for the logistic regression model than the artificial neural network model on the validation data (Supplementary Figure S1).

### Clinical app implementation

The artificial neural network model was implemented in the Re-Admit app (Supplementary Figure S2). This smartphone application communicates with the clinical data repository. The four input parameters are automatically converted into the proper inputs for the artificial neural network model, and the percentile rank of the risk score calculated by the model is then instantly returned to the interface of the app.

### Clinical intervention results

**Figure 3:**
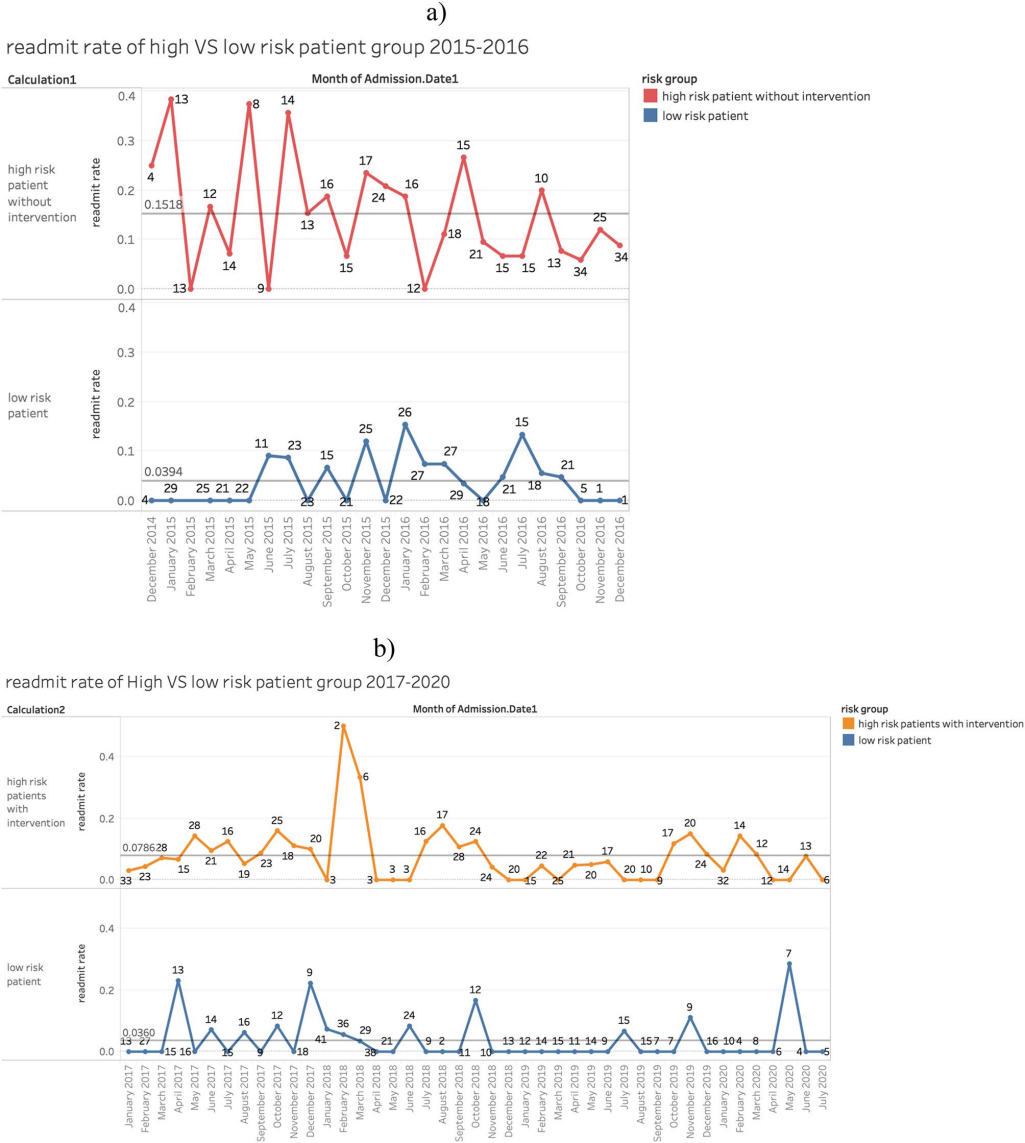
Comparisons of readmission rate on high-risk versus low-risk patients’ group before and after intervention. (**a**) The readmission rate in the high-risk patient group in 2015–2016 before intervention is 15.2% and in the low-risk patient group in 2015–2016 before intervention is 3.9%. The high-risk patients are identified by the Re-Admit App with risk scores larger than 50%. (**b**) The readmission rate in the high-risk patient group in 2017-July 2020 after intervention is 7.9% and in the low-risk patient group in 2015–2016 before intervention is 3.6%. The high-risk patients are identified by the Re-Admit App with risk scores larger than 50%.

**Figure 4:**
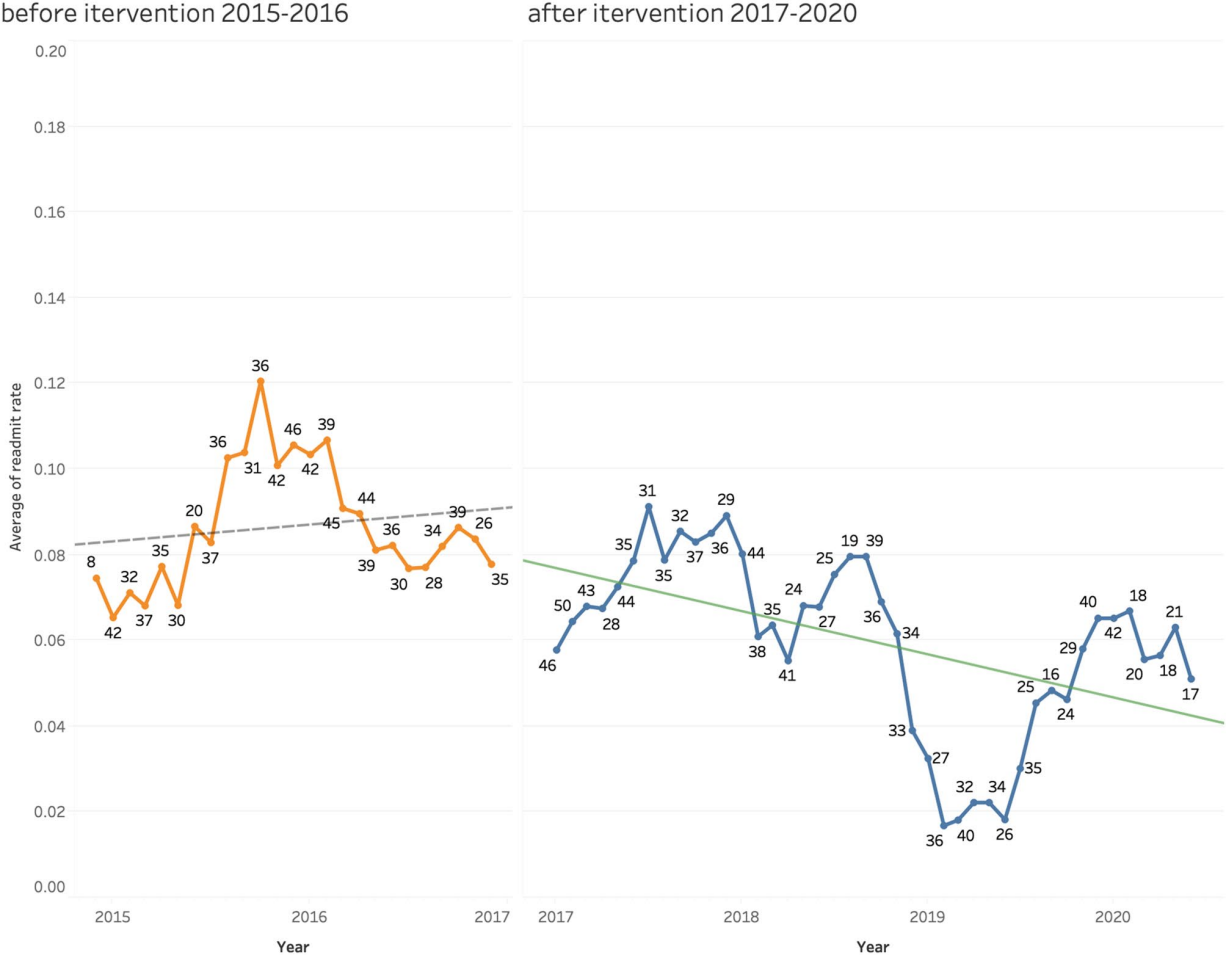
The trend of readmission rate from 2015-July 2020. The trend line was smoothed by averaging the readmission rate of 6 months. The regression lines show the readmission rate increased slightly in 2015–2016 and a decreasing trend since 2017 when the follow-up care plan intervention started.

Based on data obtained from VIZIENT (Irving, Texas, U.S.A.), among 847 COPD patient admissions from January 2015 to December 2016 before the app and intervention plan, there were 73 readmissions within 30 days (total readmission rate of 8.6%). Of 1778 patient admissions between January 2017 and July 2020 who were evaluated by Re-Admit app and given intervention according to their scores, there were 111 30-day readmissions (total readmission rate of 6.2%). Figure 3 shows the detailed readmission rates in high- and low-risk groups in each month from January 2015 to July 2020. In the low-risk group of 2015–2016 (before app and intervention), the average readmission rate was 3.9% while in the high-risk group, the average readmission rate was 15.2%. Post app and intervention i.e., January 2017–July 2020, the average readmission rate in the low-risk group was 3.6% (no significant difference with 3.9%), while in the high-risk patient group identified by the app, and subsequently receiving in-hospital clinical interventions as described, the average readmission rate reduced to 7.9%, a 48% decrease. Figure 4 shows the readmission rate trend from January 2015 to July 2020.

## Discussion

In this study, we constructed a neural network model for predicting readmission risk in patients with COPD on admission day one. The algorithm, deployed in a smartphone application, allowed the COPD Readmission Prevention Committee at Houston Methodist Hospital to predict readmission risk using only four variables conveniently and accurately. Previously published patient readmission prediction tools required multiple variables, relied on discharge parameters^10^, or were simply unreliable^11^. This risk assessment model appropriately identified high readmission risk patients with COPD. Clinical interventions successfully reduced 30-day readmissions in COPD.

Previous comparisons of logistic regression and artificial neural network models applied to patient readmission prediction have varied considerably, possibly reflecting different primary admission diagnoses^12–15^. This study found that the artificial neural network model has better predictive performance for patient readmission risks on the first day of patient admission than logistic regression models. The neural network, which can be used to simulate complex nonlinear relationships, has a more sophisticated modeling structure than logistic regression. Thus, few assumptions are required before constructing the model. Furthermore, the neural network can continue to increase in efficacy as new datasets are added.

The Re-Admit app also leverages the composite Rothman Index as one of its prediction factors of readmission likelihood. The index has been validated for assessing in-hospital mortality risk and subsequent post-discharge one-year mortality. It is widely utilized in many hospital systems, and routinely available from day one and throughout the patient’s hospital stay. However, it has not been used in any other readmission risk prediction models to date. The assessment index’s expanding acceptance and integration into the EMR of health systems nationwide and beyond translates to ease of adoption and deployment of Re-Admit in the future. We are investigating a small subset of parameters in the Rothman Index to construct the artificial neural network model when the Rothman Index is not available.

An advantage of the Re-Admit predictive model is the utilization of a small set of input parameters—four variables obtained on the first day of admission, compared to similar models requiring as many as twenty variables for similar predictive capability^16^. Existing methods for readmission prediction require information not yet available upon admission, e.g., length of hospital stay—hence the prediction can only be made on or after discharge—often too late for meaningful intervention. By identifying at-risk patients on day one of hospital admission, discharge planning/out-patient transition resources can be focused on those at greatest risk of readmission. This early intervention may reduce costs and lead to better patient outcomes for patients with COPD.

### Limitations & strengths

This study was performed on the derivative population in the Houston Methodist System (a large urban eight-hospital system) and the region (Urban Southwest US), and the generalization of its findings are needed to validate in other health systems of different patient demographics or clinical practice. Even if the Houston Methodist’s Re-Admit app is not immediately generalizable to other hospitals and systems, the artificial neural network is trainable such that the four variables could provide the same degree of readmission risk prediction that is unique to that institution or region. A disadvantage of the artificial neural network or its variants is that it needs more coefficients for training than logistic regression models. The training requirement is more demanding. Meanwhile, those variables that were significant in our patient population may or may not be relevant in other groups, thus ongoing prospective validation in other health systems is necessary.

One limitation is using the Rothman Index as this may restrict the generalization of the model. Many other hospitals may use other severity index products rather than the Rothman Index. We can replace the commercial Rothman index that is not available in the local hospital with similar index used in the electronic medical record (EMR) of the local hospital. We could also create our own indicator that performs the same function as Rothman Index using patient data in the EMR. Since we use EPIC in our health system, we plan to experiment and compare with the EPIC risk score with the purpose of using the EPIC risk score as a replacement. For generalization, a few regulatory steps need to be conducted, including: (1) obtaining the approval by IRB, (2) securing the support and engage the Readmission Risk Committee, (3) validating the Re-Admit app in both retrospective and prospective studies, (4) submitting the results to Systems Quality Control Committee for approval for use in routine clinical operation, (5) integrating into the electronic health record system of the local hospital, and (6) implementing the process to certify the Re-Admit app on an annual basis using criteria defined by the Systems Quality Control Committee or Council.

The choice of interventions to mitigate readmission included specialist notification of risk status and formal pulmonary consultations. Pharmacist and respiratory therapist training, medication reviews and interventions, early post discharge clinic/office appointment and home health care coordination were executed weekly during concurrent review of inpatients by the COPD Readmission Reduction Committee. CONNECT phone calls, an automated outreach telefonic program, were also instituted to query the patients’ needs, concerns and questions immediately post discharge.

## Conclusion

In summary, this Re-Admit app utilizing an artificial neural network-based predictive model successfully classified patients at risk for readmission with the primary diagnosis of COPD. The risk stratification was performed accurately on day one of the admission thus successfully stratifying the patients early for interventions. Focused in-hospital teaching and care-transition initiatives based on the high risk of readmission identified patients on day one. This has led to decreased 30-day readmissions for our COPD patients, improving outcomes and health care savings. This research also illustrates the emerging paradigm of the smart optimization of clinical care pathway driven by rigorously validated AI apps to improve outcomes.

## Methods

### Data collection and statistical analysis

Medical records of patients with COPD as a primary diagnosis were queried from METEOR (Methodist Environment for Translational Enhancement and Outcomes Research) clinical data warehouse of all eight Houston Methodist Hospitals^17^. As the Rothman index score is not stored as raw data, the score was manually extracted from the monitoring panel. The Rothman Index (PeraHealth, Charlotte NC) is a regularly updated integrated health score using a range of twenty-six physiological measures, including lab test results, vital signs, and nursing assessments^18^. It is an automated, proprietary third-party algorithm embedded within commercial electronic medical record systems. The Rothman index has been shown in multiple trials to be a valuable metric for predicting mortality in hospitalized patients. It has not, however, been utilized as a component for predicting readmission. Supplementary Figure S3 shows the twenty-six variables and the corresponding Rothman Index for a patient in the monitoring panel.

The following available parameters that were considered to potentially impact readmission included demographics, index admission type, day one data on severity of illness, comorbidities, laboratory data, medications on admission, Rothman Index on admission, procedures, and chief complaint for admission^19,20^. Univariate analysis was employed for each parameter to assess its association with subsequent readmission^21^. A Wald test from logistic regression was conducted on the parameters and the parameters were ranked by p-value. Parameters highly predictive of subsequent readmission were identified from the training sample, and subsequently validated against a second population of patients with COPD.

All aspects of this study were carried out in accordance with relevant guidelines and regulations including preserving patients' privacy. The Houston Methodist Hospital Institutional Review Board approved this study, and this study is one of several process quality improvement projects commissioned by Houston Methodist Hospital management to improve quality of patient care. Retroactive patient data accruals was used in developing the model and the Houston Methodist IRB granted a waiver for patient’s informed consent.

### Model building and training

The task of predicting early patient readmissions was formulated as a binary classification task—Readmission yes/no into any of Houston Methodist system hospitals within 30 days. Two mathematical modeling approaches were constructed: logistic regression^22,23^ and artificial neural network^24,25^.

### Training and testing the neural network

Artificial neural network has the advantage of modeling complex nonlinear functions. A classic neural network includes an input layer, some hidden layers, and an output layer. Each layer contains some nodes or neurons. Each node in the hidden layer is a mathematical function to transfer information from input to output. Connections between two nodes from two adjacent layers are called weights. The logistic regression model is considered as a simple form of the neural network with only one node in the hidden layer and one output unit. A model of components in a simple neutral network is presented in Supplementary Figure S4.

The artificial neural network can be described by mathematical formula as:

$$y_{i}^{\left( 1 \right)} = f\left( {\mathop \sum \limits_{j = 1}^{{m_{0} }} w_{i,j}^{\left( 1 \right)} *y_{j}^{\left( 0 \right)} + w_{0,j} } \right)$$

whereas ${y}_{j}^{\left(0\right)}$ is the *j*th input in the input layer, ${w}_{i,j}^{\left(1\right)}$ the connection weight from the *j*th input node to the *i*th node in the first hidden layer, ${w}_{0,j}$ the bias, and $f(x)$ function referred as activation function which is some predefined function, such as the hyperbolic tangent, sigmoid function, softmax function, or Gaussian function. The information of each layer is passed to the next layer based on the formula until the output layer.

The best artificial neural network model was settled using a three-fold cross-validation method; the same procedure was conducted to train the logistic regression model for comparison proposes. For each round of three-fold cross-validation, the neural network model was trained a hundred times with different randomly initialized coefficients, as the neural network is easily trapped into the local optimal solution because of improper initialization. The network with the best prediction performance with the test data set was selected as the best model for each round. The final performance was estimated on the average performance of three-fold cross-validation. That process was repeated four times with 2, 3, 4, or 5 nodes in the hidden layer with the purpose of determining the optimal number of hidden nodes. After the number of nodes settled, we compared the artificial neural network model with the logistic regression model based on their average performance of three-fold cross-validation. Furthermore, the output scores of the final neural network model on the whole training samples were converted to percentiles as the final risk score for readmission. Percentiles equal to or greater than 50% are considered high risk for readmission.

The training of the neural network prediction model was conducted by using the ‘neuralnet’ package^26^ in R^27^. The neural network was trained by the resilient backpropagation (RPROP)^28^ with weight backtracking method.

### Validation of the models

For validation purposes, the logistic regression and artificial neural network algorithms were applied prospectively to predict readmission in COPD patients admitted from separate time periods. The performance of prediction of readmissions was then compared between the neural network model and logistic regression model. Area under the curve of receiver operating characteristic (AUCROC) curves, sensitivity, specificity, and positive predictive value (PPV) were calculated to compare the readmission prediction in the two models. Model calibration was evaluated using plots of predicted versus observed 30-day COPD readmission rate.

### Implementation of high readmission risk protocols for COPD cohort

Since 2017, patients with a primary diagnosis of COPD were evaluated weekly by the hospital’s Readmissions Reduction Committee, which reviewed the patients’ diagnosis and care plan in the context of the readmission risk as determined by the predictive model. Patients identified as high risk for readmission received the following: specialist consultation or notification of risk status, medical educational visits by clinical pharmacists and respiratory therapists, and home health and early physician follow-up visits scheduled prior to discharge. Implementation of transition telephone calls following discharge (CONNECT) ensured that all aspects of discharge planning were proceeding properly.

All COPD patients admitted between January 2015 and July 2020 from VIZIENT were evaluated to compare readmission rates before and after the introduction of app prediction and subsequent interventions. VIZIENT is an external dataset providing readmission data inclusive of readmissions outside the Houston Methodist system. Ultimately, the Re-Admit app is made available on the smartphones of clinicians at the bedside.

### Statistical analysis

Model performance was evaluated based on the AUC with standard variance, and 95% CIs of sensitivity, specificity, and positive predictive value (PPV). The average AUC of the models on the training set was computed on the threefold cross-validation. 95% CIs of the metrics for the neural network model on the validation dataset was computed with 2000 bootstrapping. Calibration analysis was performed to compare the alignment for the logistic regression model and the artificial neural network model on the validation data.

## Supplementary Information

Supplementary Information.

## Data Availability

The datasets analyzed during the current study are not publicly available. Due to privacy and security concerns, the EHR data are not redistributable to researchers other than those engaged in this study.

## Code availability

Code for the algorithms used for analyzing the EHR data; development and evaluation the model; and statistical analysis are available from the corresponding author upon reasonable request.

## References

[1] Jencks SF, Williams MV, Coleman EA (2009). Rehospitalizations among patients in the Medicare fee-for -service program. N. Engl. J. Med..

[2] 2017 Condition-Specific Measures Updates and Specification Report Hospital –Level 30 –Day Risk-Standardized Readmission Measures. Yale New Haven Health Services Corporation/Center for Outcomes Research and Evaluation, Center for Medicare & Medicaid Services (CMS) March 2017.

[3] Zuckerman RB, Sheingold SH, Orav EJ, Ruhter J, Epstein AM (2016). Readmissions, observation, and the Hospital Readmissions Reduction Program. N. Engl. J. Med..

[4] Mansukhani RP, Bridgeman MB, Candelario D, Eckert LJ (2015). Exploring transitional care: evidence-based strategies for improving provider communication and reducing readmissions. P. T..

[5] Finlayson K, Chang AM, Courtney MD, Edwards HE, Parker AW, Hamilton K (2018). Transitional care interventions reduce unplanned hospital readmissions in high-risk older adults. BMC Health Serv. Res..

[6] Kansagara D, Englander H, Salantro A, Kagen D, Tehobal C, Freeman M, Kripalani S (2011). Risk prediction models for hospital readmission: a systematic review. JAMA.

[7] Zhou H, Della PR, Roberts P, Goh L, Dhaliwal SS (2016). Utility of models to predict 28-day or 30-day unplanned hospital readmissions: an updated systematic review. BMJ Open.

[8] García S, Luengo J, Sáez JA, López V, Herrera F (2013). A survey of discretization techniques: Taxonomy and empirical analysis in supervised learning. IEEE Trans. Knowl. Data Eng..

[9] Beck MW (2018). NeuralNetTools: Visualization and analysis tools for neural networks. J. Stat. Softw..

[10] Burke RE, Schnipper JL, Williams MV, Robinson EJ, Vasilevskis EE, Kripalani S, Metlay JP, Fletcher GS, Auerbach AD, Donze JD (2017). The HOSPITAL Score predicts potentially preventable 30 day readmission in conditions targeted by the hospital readmission reduction program. Med. Care.

[11] Bashir B, Schneider D, Naglak MC, Churilla TM, Adelsberger M (2016). Evaluation of prediction strategy and care coordination for COPD readmission. Hosp. Pract..

[12] Francis NK, Luther A, Salib E, Allanby L, Messenger D, Allison AS, Smart NJ, Ockrim JB (2015). The use of artificial neural networks to predict delayed discharge and readmission in enhanced recovery following laparoscopic colorectal cancer surgery. Tech. Coloproctol..

[13] Kulkarni P, Smith LD, Woeltje KF (2016). Assessing risk of hospital readmissions for improving medical practice. Health Care Manag. Sci..

[14] Ottenbacher KJ, Smith PM, Illig SB, Linn RT, Fiedler RC, Granger CV (2001). Comparison of logistic regression and neural networks to predict rehospitalization in patients with stroke. J. Clin Epidemiol..

[15] Lee FW (2012). Selecting the best prediction model for readmission. J. Prev. Med. Public Health.

[16] Lau C, Siracuse BR, Chamberlain RS (2017). Readmission after COPD exacerbation scale: determining 30-day readmisison risk for COPD patients. Int. J. Chron Obstuct. Pulmon. Dis..

[17] Puppala M, He T, Chen S, Ogunti R, Yu X, Li F, Jackson R, Wong ST (2015). METEOR: An enterprise health informatics environment to support evidence-based medicine. IEEE Trans. Biomed. Eng..

[18] Rothman MJ, Rothman SI, Beals J (2013). Development and validation of a continuous measure of patient condition using the electronic medical record. J. Biomed Inform..

[19] Ben-Chetrit E, Chen-Shuali C, Zimran E, Munter G, Nesher G (2012). A simplified scoring tool for prediction of readmission in elderly patients hospitalized in internal medicine departments. Isr. Med. Assoc. J..

[20] Bowles K, Chittams J, Heil E, Topaz M, Rickard KA, Bhasker M, Tanzer M, Behta M, Hanlon A (2015). Successful electronic implementation of discharge referral decision support has a positive impact on 30- and 60-day readmissions. Res. Nurs. Health.

[21] Bursac, Z., Gauss, C. H., Williams, D. K. & Hosmer, D. W. Purposeful selection of variables in logistic regression. *Source Code Biol. Med.* 3–17 (2008). 10.1186/1751-0473-3-17 PMC2633005 19087314

[22] Zhou X, Liu KY, Wong ST (2004). Cancer classification and prediction using logistic regression with Bayesian gene selection. J. Biomed. Inform..

[23] Kleinbaum DG, Klein M (2010). Logistic Regression—A Self-Learning Text.

[24] Hammoudi, A. A. *et al.* Automated Nuclear Segmentation of coherent anti-stokes raman scattering microscopy images by coupling superpixel context information with artificial neural networks. *MLMI*, 317–325 (2011).

[25] Da Silva IN, Spatti DH (2016). Artificial Neural Networks: A Practical Course.

[26] Fritsch S, Guenther F. neuralnet: Training of Neural Networks. R package version 1.33. https://CRAN.R-project.org/package=neuralnet

[27] R: https://www.r-project.org/certification.html

[28] Riedmiller M, Braun H. A direct adaptive method for faster backpropagation learning: The RPROP algorithm. In *Proceedings of International Conference on Neural Networks*, 586–591 (1993).

