# Supplementary material for: An AI-driven clinical care pathway to reduce 30-day readmission for chronic obstructive pulmonary disease (COPD) patients
Source: Sci Rep. 2022 Nov 30;12:20633. doi: 10.1038/s41598-022-22434-3 (PMC9712389; doi:10.1038/s41598-022-22434-3)
Supplement: Supplementary file 1 — Supplementary Information. [file 41598_2022_22434_MOESM1_ESM.docx]

**Supplemental Table S1.** Univariate analysis result for each parameter of COPD patients. The statistical tests used for the univariate analysis and corresponding P values are listed in the last two columns. ANOVA: ANOVA test; t-test: Student's t-test; Fisher test: Fisher’s exact test.

| Variable | Readmit | | p value |  |
| --- | --- | --- | --- | --- |
|  | yes | no |  |  |
| Age in years  Mean (± sd) | 69.0(11.9) | 68.4(12.2) | 0.315 | ANOVA |
|  |  |  | 0.306 | t-test |
| Sex *(*%) |  |  | 0.0891 | ANOVA |
|  |  |  | 0.0927 | Fisher test |
| *Female* | 57.0 | 61.0 |  |  |
| *Male* | 43.0 | 39.0 |  |  |
| Race (%) |  |  | 0.0148 * | ANOVA |
|  |  |  | 0.01479 * | Chi-square |
| *Asian* | 1.9 | 1.8 |  |  |
| *Black* | 26.7 | 22.0 |  |  |
| *Caucasian* | 66.1 | 68.5 |  |  |
| *Hispanic* | 0.4 | 0.4 |  |  |
| *Indian, America* | 0.2 | 0.08 |  |  |
| *Other* | 4.7 | 7.3 |  |  |
| Admission Source (%) |  |  | 0.325 | ANOVA |
|  |  |  | 0.325 | Chi-square |
| *Clinic or Phys Office* | 7.0 | 5.7 |  |  |
| *Emergency Room Dis* | 69.0 | 70.0 |  |  |
| *Non-Healthcare Facility* | 19.6 | 21.0 |  |  |
| *Other* | 4.5 | 3.3 |  |  |
|  |  |  |  |  |
| Discharge Disposition (%) |  |  | 0.0408 * | ANOVA |
|  |  |  | 0.04625 * | Fisher test |
| *Routine Discharge Home* | 65.5 | 70.1 |  |  |
| *Other* | 34.5 | 29.9 |  |  |
| Risk of Mortality  Mean (± sd) | 2.45 (0.87) | 2.26 (0.94) | 2.11e-05 *** | ANOVA |
|  |  |  | 8.99e-06 *** | t-test |
| Severity of Illness  Mean (± sd) | 2.72 (0.79) | 2.50(0.83) | 3.49E-08 *** | ANOVA |
|  |  |  | 1.826e-08 *** | t-test |
| Length of Stay (LOS)  Mean (± sd) | 5.93(4.79) | 4.99(4.57) | 2.87E-05 *** | ANOVA |
|  |  |  | 2.87E-05 *** | t-test |
| Number of diagnosis POA Mean (± sd) | 11.79 (4.97) | 10.29 (4.67) | 6.13E-11 *** | ANOVA |
|  |  |  | 5.173e-10 *** | t-test |
| Payor (%) |  |  | 0.00656 ** | ANOVA |
|  |  |  | 0.006611 ** | Chi-square |
| *Medicare* | 77.7 | 71.4 |  |  |
| *Managed Care* | 8.5 | 13.4 |  |  |
| *Other* | 2.7 | 1.9 |  |  |
| *Self-pay* | 2.9 | 3.6 |  |  |
| *Unknown* | 8.1 | 9.6 |  |  |
|  |  |  |  |  |
| Last 6 months Inpatient visits  Mean (± sd) | 1.37 (1.70) | 0.71(1.20) | <2e-16 *** | ANOVA |
|  |  |  | <2e-16 *** | t-test |
| # unique medications administered on the day of admission  Mean (± sd) | 11.03 (5.95) | 10.55(5.68) | 0.0825 | ANOVA |
|  |  |  | 0.09222 | t-test |
| Marital Status |  |  | 0.000118 *** | ANOVA |
|  |  |  | 0.000118 *** | Fisher test |
| Last 6 months # of unique medications  mean (± sd) | 25.58721(21.60668) | 17.12824(17.10666) | <2e-16 *** | ANOVA |
| Last 6 months # of unique diagnosis | 31.47868 (18.88611) | 23.10356 (15.28231) | <2e-16 *** | ANOVA |

| **Supplemental Table S2.** Characteristics of COPD patients. | |
| --- | --- |
| Total number | 3005 |
| Age  Mean (± sd) | 68.53(± 12.18) |
| Sex (%) |  |
| *Female* | 60.31% |
| *Male* | 39.69% |
| Race (%) |  |
| *Asian* | 1.84% |
| *Black* | 22.79% |
| *Caucasian* | 68.07% |
| *Hispanic* | 0.37% |
| *Indian, America* | 0.10% |
| *Other* | 6.83% |
| Admission Source (%) |  |
| *Clinic or Phys Office* | 5.9% |
| *Emergency Room Dis* | 70.0% |
| *Non-Healthcare Facility* | 20.6% |
| *Other* | 3.5% |
| Discharge Disposition (%) |  |
| *Routine Discharge Home* | 69.7% |
| *Other* | 30.3% |
| Length of Stay (LOS)  Mean (± sd) | 5.15(± 4.62) |
| Number of diagnosis POA  Mean (± sd) | 10.61(± 4.72) |

**Supplemental Table S3.** Relative importance of the input variables evaluated by Garson’s algorithm.

| Variables sort by relative importance in the neural network model. |
| --- |
| 1) Insurance status: Self Pay,  2) Number of in-patient visits within prior 6 months,  3) Rothman Index at the day of admission,  4) number of unique medications at admission day,  5) Insurance status: Medicare,  6) Insurance status: MGD Care,  7) Insurance status: Unknown,  8) Insurance status: Other |

**Supplemental Figure S1.**  Calibration Plots for Validation data for LG and ANN Models. Left: calibration curve for LG model; right: calibration curve for ANN Model.

**Supplemental Figure S2:** The entry screen and history screen of the HM Re-Admit App.

**
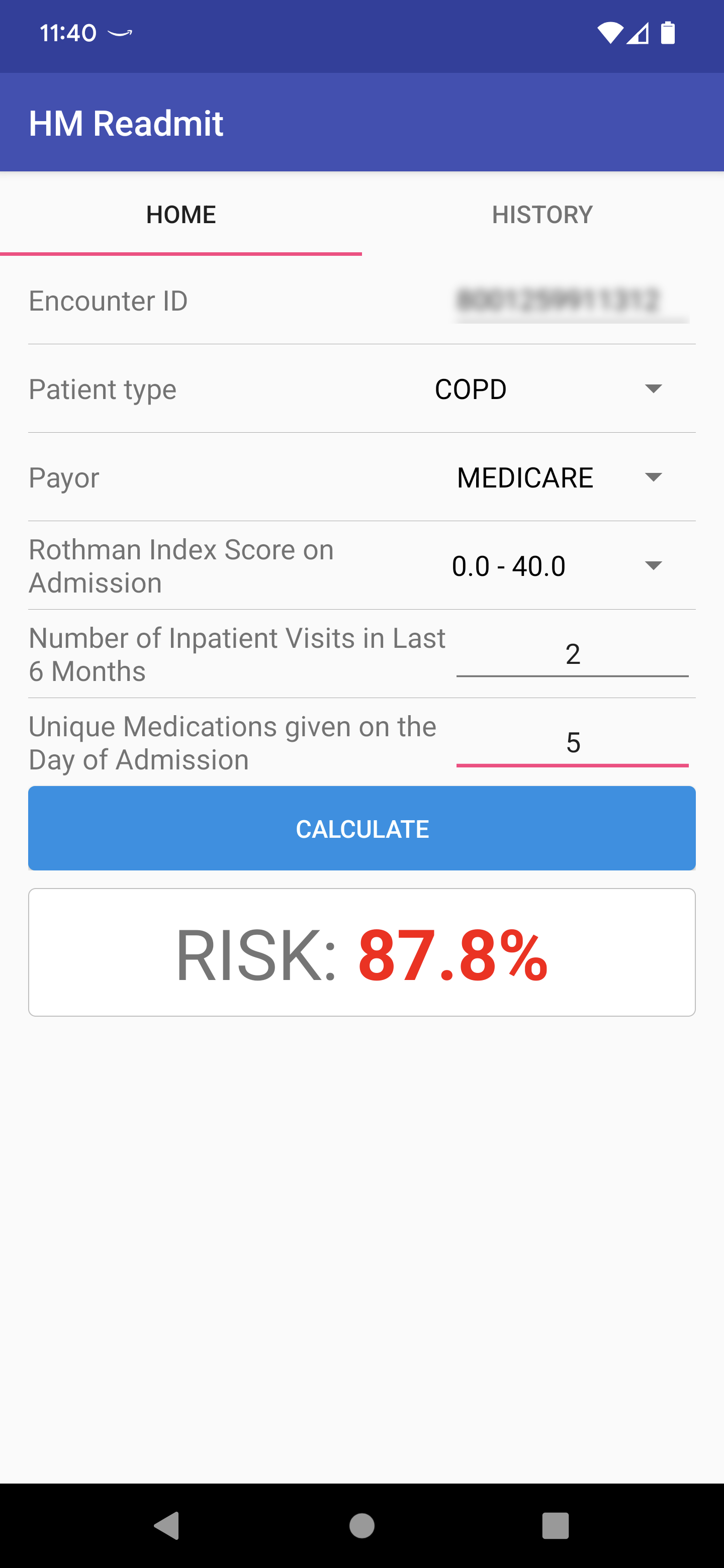
** **
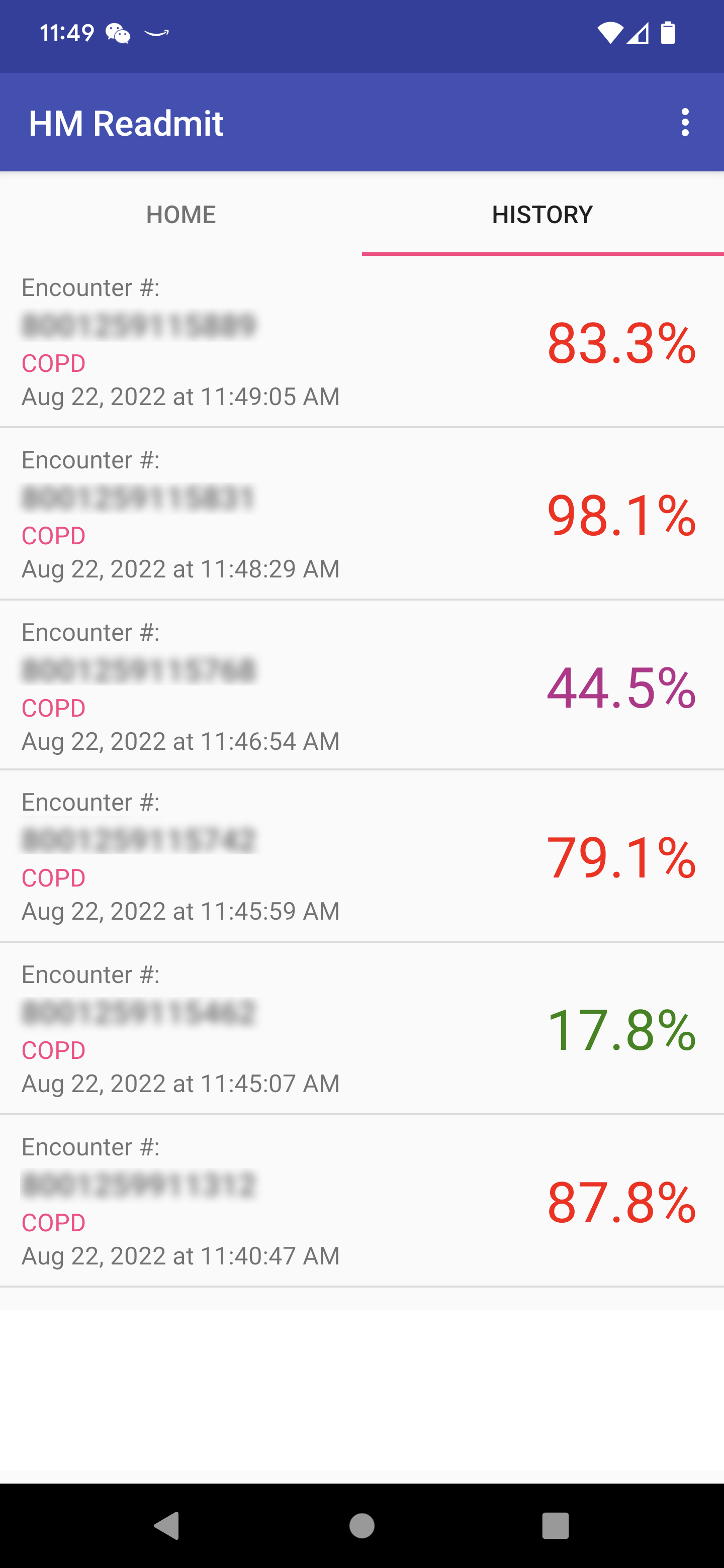
**

**Supplemental Figure S3:** The Rothman Index Score may use 26 variables, for example, vital signs, including temperature, systolic blood pressure, diastolic blood pressure, heart rate, blood oxygen saturation and respiratory rate; nursing assessments, including cardiac, food/nutrition, gastrointestinal, genitourinary, musculoskeletal, neurological, peripheral vascular, psycho-social, respiratory, skin/tissue and safety/fall risk standards; scores, including Braden Scale Heart patterns; blood chemistry, including blood urea nitrogen, creatinine, chloride, sodium and potassium; and blood analysis, including hemoglobin and white blood cell count. A patient example with 26 variables and corresponding Rothman Index.


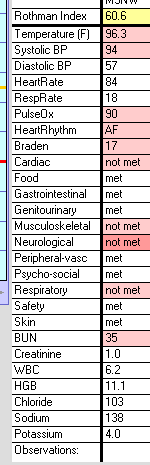


**Supplemental Figure S4:** An example of ANN with input, hidden and output layers.

Input layer

Hidden layer

Output Layer
